# Worms in the Bladder

**Published:** 1833

**Authors:** Leonard Bardwell

**Affiliations:** Pendleton, Indiana


### Art. V.—Worms in the Bladder.

*An account of a case in which a large number of Worms were discharged from the Bladder and the Bowels. By* Dr. Leonard Bardwell.

A man aged 30 years, applied to Dr. Cook and myself, in the month of August, 1832, for medical aid. He was an emigrant from the south-east of North Carolina, where he had been subject to intermittent fever, from his infancy up to manhood. For the few last years he had followed the sea, and while on a voyage to the E. Indies, was attacked with a painful and peculiar sensation in the region of the bladder. His digestive functions soon became much impaired, and his general health declined. The pain increased sometimes to perfect agony, attended with difficulty in urinating. He consulted a number of medical men, who told him his case was stone, and he was under that impression when he consulted us. Adopting the same opinion, we advised, in succession, various lithontriptics, with little or no benefit. Then chylopoietic alteratives and tonics, with similar bad success. By this time he had begun to complain of pain in the bowels and region of the liver, with some difficulty of breathing. A vague idea that he had worms, came into the mind of one of us, and we determined to try a vermifuge. Accordingly, we gave an active dose of calomel at night, and directed him to eat nothing but gruel, which brought away nineteen large worms, near twelve inches long. On the night following, he took one ounce of castor oil, with the same quantity of spirits turpentine; and, before day, discharged between 70 and 80 more, of the same appearance. During the operation of the oil, he felt strongly disposed to make water, but could not discharge even a drop; and the distress in the bladder became intolerable, attended with the peculiar kind of sensation, such as heat, tickling, itching, or gnawing, such as he had experienced before, and indeed first suggested to us that he might possibly have worms. After daylight, he called for the vessel, to make water; and commenced discharging bloody urine and small worms, which came so thick as to require pulling away. He certainly discharged, at that time, at least two thousand, which gave great relief. The oil and turpentine were continued until many thousands must have been evacuated. Meanwhile his health rapidly improved, though he continued to discharge worms of a similar kind, for six months afterwards. He is now perfectly healthy.

The worms which came from the bladder, were from onehalf to three-fourths of an inch in length; about the thickness of a very large horse hair, and of a dark, dirty, white color, with a black head, rather large in size. They possessed considerable activity, could crawl on the ground, or on a board; and many of them lived 24 hours after they were discharged, and were seen by numerous persons. I am sorry that we had not preserved a specimen of them.

*Pendleton, Indiana, Nov.* 1833.

